# Current evidence on the relationships among five polymorphisms in the matrix metalloproteinases genes and prostate cancer risk

**DOI:** 10.1038/s41598-024-62016-z

**Published:** 2024-05-18

**Authors:** Jiandong Gui, Hangsheng Zhou, Sixin Li, Anjie Chen, Qing Liu, Lijie Zhu, Yuanyuan Mi

**Affiliations:** 1 Wuxi School of Medicine, Jiangnan University, 1800 Lihudadao, Wuxi, 214122 Jiangsu Province China; 2 Department of Urology, Affiliated Hospital of Jiangnan University, 1000 Hefeng Road, Wuxi, 214122 Jiangsu Province China; 3 Huadong Sanatorium, 67 Dajishan, Wuxi, 214122 Jiangsu Province China

**Keywords:** Prostate cancer, Matrix metalloproteinases (MMPs), Polymorphism, Tumor marker, Meta-analysis, Cancer, Genetics, Oncology, Urology

## Abstract

Matrix metalloproteinases (MMPs) had a variety of subtypes, which may be related to tumor invasion and angiogenesis, and the polymorphisms from MMPs have been also associated with the susceptibility to a variety of tumors, including prostate cancer (PCa). However, previous studies have not systematically analyzed the association between MMP and prostate cancer, so we conducted systematic data collection and analyzed to evaluate the relationship among polymorphisms in MMPs and PCa susceptibility. We searched PubMed, Web of Science, Embase and Google Scholar for all papers published up to Apr 3rd, 2023, and systematically analyzed the relationship among MMP1-1607 2G/1G, MMP2-1306 T/C, MMP2-735 T/C, MMP7-181 G/A, MMP9-1562 T/C and PCa susceptibility using multiple comparative models and subgroup analyses. We found that MMP2-1306 T/C polymorphism showed associations with PCa susceptibility, with the Ethnicity subgroup (Asian) being more pronounced. Similarly, MMP9-1562 T/C has also had associations with PCa susceptibility. Our current study found that the polymorphisms of, MMP2-1306 T/C, and MMP9-1562 T/C had strong associations with PCa risk.

## Introduction

The incidence of prostate cancer (PCa) is increasing in developed countries. It is estimated that the number of PCa cases in men in the United States will reach 288,300, accounting for 29% of all cancers in 2023^1^. However, the incidence and mortality rates of cancer in China are changing from a developing country to a developed country, and the incidence of PCa is increasing year by year^2^.

PCa is difficult to diagnose early, and studies have found that PCa may be related to family history, race, occupation, cadmium exposure, vasectomy, diet, hormones and other factors, and genetic factors may be one of the most important factors^3^. It is considered important to obtain the family history of all cancer patients and to screen patients with genetic cancer susceptibility, but there is currently a lack of available families with genetic susceptibility^4^.

MMPs are a multigene family of zinc-dependent endopeptidases that share similar structures and share the ability to degrade virtually all components of the extracellular matrix (ECM), MMPs playing a central role in morphogenesis, wound healing, tissue repair, and remodeling to deal with injuries^5^. The degradation products of ECM have unique biological properties, which can promote various tumor processes^6^. MMP11 and MMP13 are almost universally upregulated in cancer, and many MMPs subtypes have potential as cancer markers^7^. The relationship between MMPs and PCa has also been continuously studied. Some studies have also shown that MMPs is correlated with Gleason score, disease-free survival, tumor recurrence and other factors of PCa^8–10^. Increased expression of MMP-2 in malignant prostatic epithelium can be an independent predictor of reduced disease-free survival of PCa^11^. MMPs can also regulate signaling pathways that control cell growth, inflammation or angiogenesis, and can even act in non-proteolytic ways^12^. Some studies even show that MMP2 and MMP9 are related to platelet aggregation, AR status, and PCa invasion. It is feasible to reduce the expression of MMPs through anticoagulation to achieve the purpose of PCa treatment^13,14^. Studying MMPs polymorphism can provide a better understanding of its functions and establish a connection between epigenetics and diseases, thereby improving preventive measures for affected individuals.

There have been 14 previous articles on the relationship between MMPs and PCa susceptibility, but the data volume of a single article is relatively insufficient, and there is inevitable heterogeneity. However, the relationship between MMPs and PCa was not clear or even contradictory in previous studies. As the number of research samples continues to expand, the total amount of data available is also constantly increasing. Based on previous data results, our study conducted systematic subgroup analysis of MMP1-1607 2G/1G, MMP2-1306 T/C, MMP2-735 T/C, MMP7-181 G/A, MMP9-1562 T/C, and added the analysis of tumor-related factors in some public databases. More comprehensive analysis results had been obtained.

## Materials and methods

### Finding and analyzing appropriate studies

Prior to Apr 3rd, 2023, we conducted a search on PubMed, Web of Science, Embase, Google Scholar and Chinese database using the keywords "matrix metalloproteinases or MMPs", "MMP1", "MMP2", "MMP7", "MMP9", "polymorphism", and "prostate cancer" to identify potentially relevant studies examining the associations among polymorphisms of MMPs and PCa risk. The references of selected studies were also examined to further screen for relevant studies. A total of 2980 articles were retrieved, with only 14^15–28^ ultimately meeting the inclusion criteria.

### Inclusion and exclusion criteria

The inclusion criteria were studies that focused on the relationship between polymorphisms of MMPs genes and PCa, employing either a case–control or cohort design, including sufficient genotype data for meta-analysis. We also excluded studies that (a) did not employ a control sample, (b) did not provide genotype frequency, or (c) family-based studies.

### Data extraction

To retrieve the data, we employed two author-based selection criteria, collecting information such as the first author's surname, publication year, polymorphisms of MMPs genes, country of origin, ethnicity of control subjects, number of case and control subjects, degree of genotyping proficiency in the control group.

### Statistics analysis

Stratification was performed based on genetic polymorphisms, with one or more studies included in each subgroup. Additionally, we identified three ethnic subgroups: Asian, Caucasian, and Mixed populations. Furthermore, the data can be categorized into two groups based on their sources: hospital based (HB) and population based (PB). MMPs genes polymorphisms and PCa risk were determined by analyzing the distribution of genotypes in the case and control groups. We performed a *Z*-test to examine the overall odds ratio (OR)^29^. Heterogeneity was assessed using chi-squared *Q*-tests, which indicated no statistically significant heterogeneity across the trials as the *P* value was greater than 0.05. To address potential significant heterogeneity, we used the random-effects model^30,31^. We also employed various genetic models such as dominant genetic model (MM + MW vs. WW), recessive genetic model (MM vs. MW + WW), homozygote comparison (MM vs. WW), allele contrast (M allele vs. W allele), and heterozygote comparison (MW vs. WW) to examine the relationship between the polymorphisms of MMPs genes and PCa risk. Furthermore, we used Pearson's chi-squared test to calculate HWE in the control group, Egger's regression test, and Begg's funnel plot to assess publication bias^32^. Lastly, we conducted the statistical analysis of our meta-analysis using Stata software version 11.0 (StataCorp LP, College Station, TX).

## Results

### Meta-analysis study characteristics

**Figure 1:**
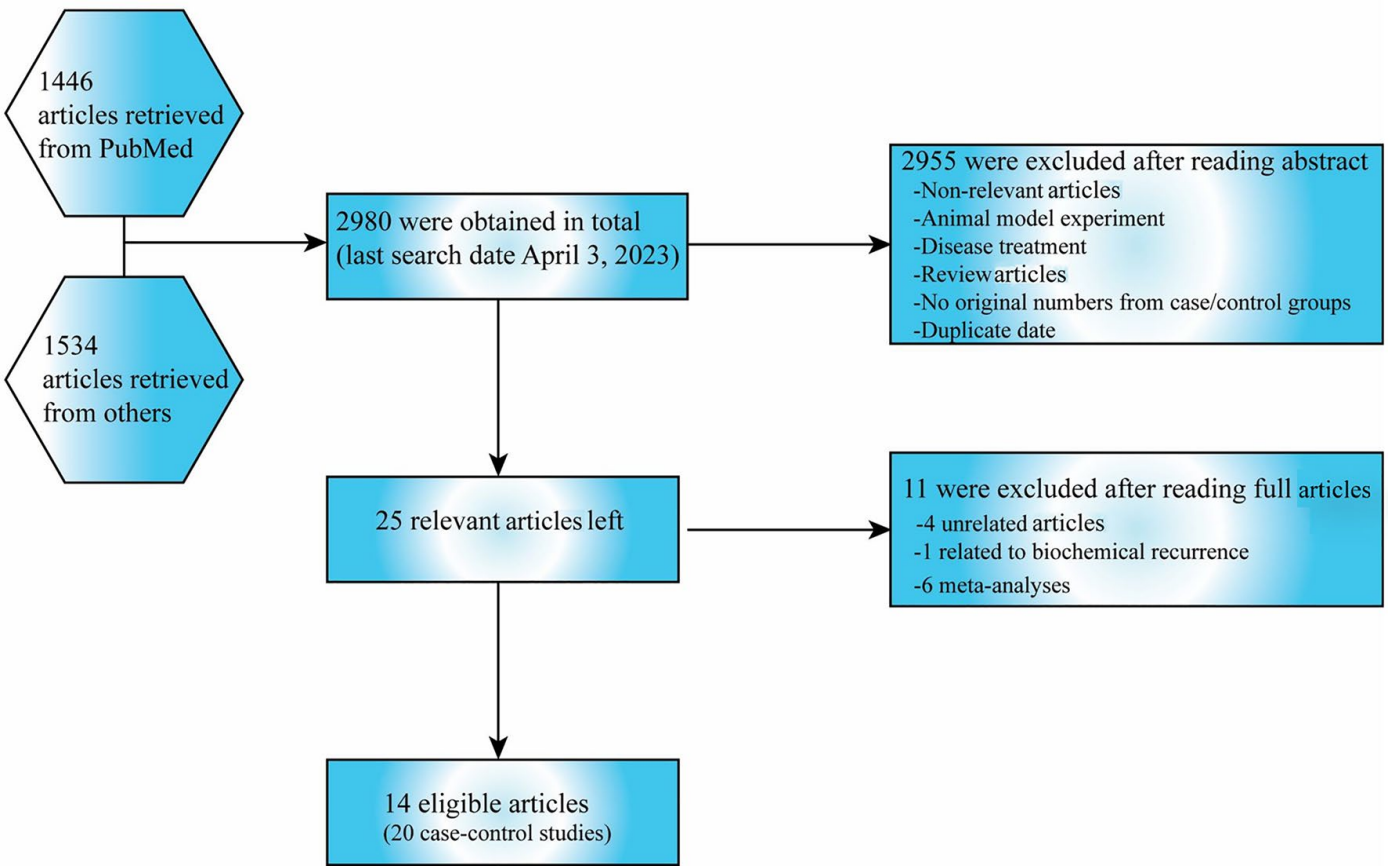
Flow chart about the search and screening strategies for the polymorphisms of MMP1-1607 2G/1G, MMP2-1306 T/C, MMP2-735 T/C, MMP7-181 G/A, MMP9-1562 T/C studies from several database.

**Figure 2:**
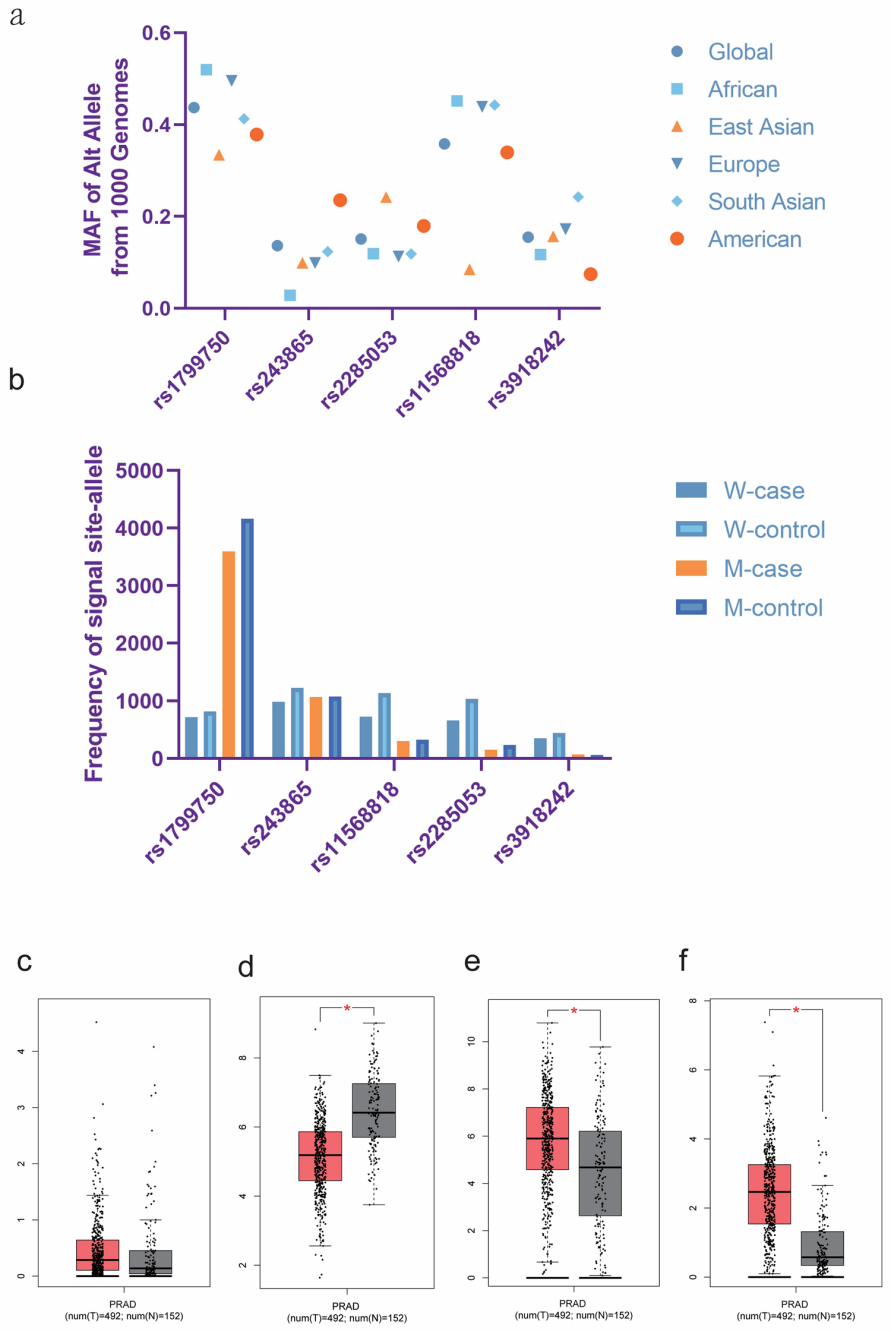
(**a**) The MAF of Alt allele for the five SNPs from the online 1000 Genome; (**b**) C- and T- allele frequencies in the case group and control group of five SNPs; (**c**-**f**) Comparison of MMPs expression levels between PRAD and normal tissues. The c-f diagram shows MMP1, MMP2, MMP7, and MMP9, respectively.

We initially identified 2980 articles from multiple databases, but after careful screening, we selected 25 distinct articles (search conducted on Apr 3rd, 2023). Subsequently, we excluded four articles that were unrelated, one article focused on biochemical recurrence, and six meta-analyses. This left us with a total of 14 articles (comprising 20 case–control studies) that met our inclusion criteria. Ultimately, we obtained 20 case–control studies that investigated the association between polymorphisms of MMPs genes and PCa risk (Fig. 1). In Supplementary Table 1, all information concerning the literature was presented, including first author, number of controls and cases, type of MMPs gene polymorphisms, year of publication, ethnicity, genotyping method, and control sources. Among the case–control studies retrieved, 3323 cases and 4043 controls were included, and the control group comprised mainly healthy people. In this study, we conducted subgroup analysis by ethnicity. Genotype counts of the analyzed polymorphisms of studies included in the meta-analysis were showed in Table 1. MAF (minor allele frequency) of five SNPs in six major populations worldwide were analyzed in 1000 Genome browsers (https://www.ncbi.nlm.nih.gov/snp) (Fig. 2a), then the frequency of signal site-allele were analyzed and compared (Fig. 2b). The comparison of the expressions of four MMPs in prostate adenocarcinoma (PRAD) and normal tissues was analyzed in Gene expression profiling interactive analysis (GEPIA), and it was found that the expression of MMP2 in normal tissues was higher than that in PRAD, while the expression of MMP7 and MMP9 in PRAD was higher than that in normal tissues, and no significant difference was observed in MMP1 (http://gepia.cancer-pku.cn/) (Fig. 2 c-f).

**Table 1:** Genotype counts of the analyzed polymorphisms of studies included in the meta-analysis.

Study	Gens	Polymorphisms	WW (cases)	WM (cases)	MM (cases)	W (%) (cases)	WW (controls)	WM (controls)	MM (controls)	W (%) (controls)	HWE
Albayrak	MMP-1	-1607 1G/2G	10	7	38	27 (25%)	7	3	33	21 (23%)	< 0.05
Sfar	MMP-9	-1562 C/T	74	25	2	173 (86%)	94	12	0	200 (94%)	0.54
dos Reis	MMP-1 MMP-2 MMP-7	-1607 1G/2G -1306 C/T -181 A/G	21 50 33	52 38 41	27 1 26	49 (32%) 107 (63%) 73 (44%)	11 59 25	34 20 39	55 21 36	29 (17%) 125 (67%) 57 (34%)	0.117 < 0.05 0.035
Jacobs	MMP-2	-1306 C/T	793	535	90	1593 (69%)	826	536	87	1659 (70%)	0.997
Tsuchiya	MMP-1	-1607 1G/2G	35	122	126	77 (17%)	33	100	118	73 (18%)	0.113
Srivastava	MMP-2	-735 C/T -1306 C/T	132 101	50 78	8 11	314 (56%) 209 (68%)	135 131	60 62	5 7	330 (83%) 269 (96%)	0.851 0.919
Yayksali	MMP-2	-1306 C/T	51	7	3	109 (90%)	42	4	0	91 (82%)	0.757
Adabi	MMP-2	-1306 C/T	74	27	0	155 (85%)	113	23	1	233 (37%)	0.884
Shajarehpoor Salavati	MMP-2	-1306 C/T	34	11	5	75 (78%)	41	7	6	89 (65%)	< 0.05
Bialkowska	MMP-1 MMP-2 MMP-7	-1607 1G/2G -1306 C/T -181 A/G	56 104 59	105 79 100	36 14 38	119 (40%) 215 (67%) 125 (42%)	54 101 76	90 78 97	53 18 24	115 (29%) 209 (65%) 159 (52%)	0.225 0.601 0.411
Liao	MMP-1	-1607 1G/2G	51	88	79	109 (31%)	96	193	147	199 (29%)	0.032
Chen	MMP-2	-735 C/T -1306 C/T	140 193	66 23	12 2	346 (79%) 409 (94%)	76 373	97 59	24 4	330 (83%) 805 (92%)	0.581 0.438
Kiani	MMP-9	-1562 C/T	72	36	4	180 (80%)	100	49	1	249 (83%)	0.053
Liao	MMP-7	-181 A/G	191	22	5	404 (93%)	372	59	5	803 (92%)	0.135

W/M: Wild/Mutant; HWE: Hardy–Weinberg equilibrium.

### Meta-analysis

**Figure 3:**
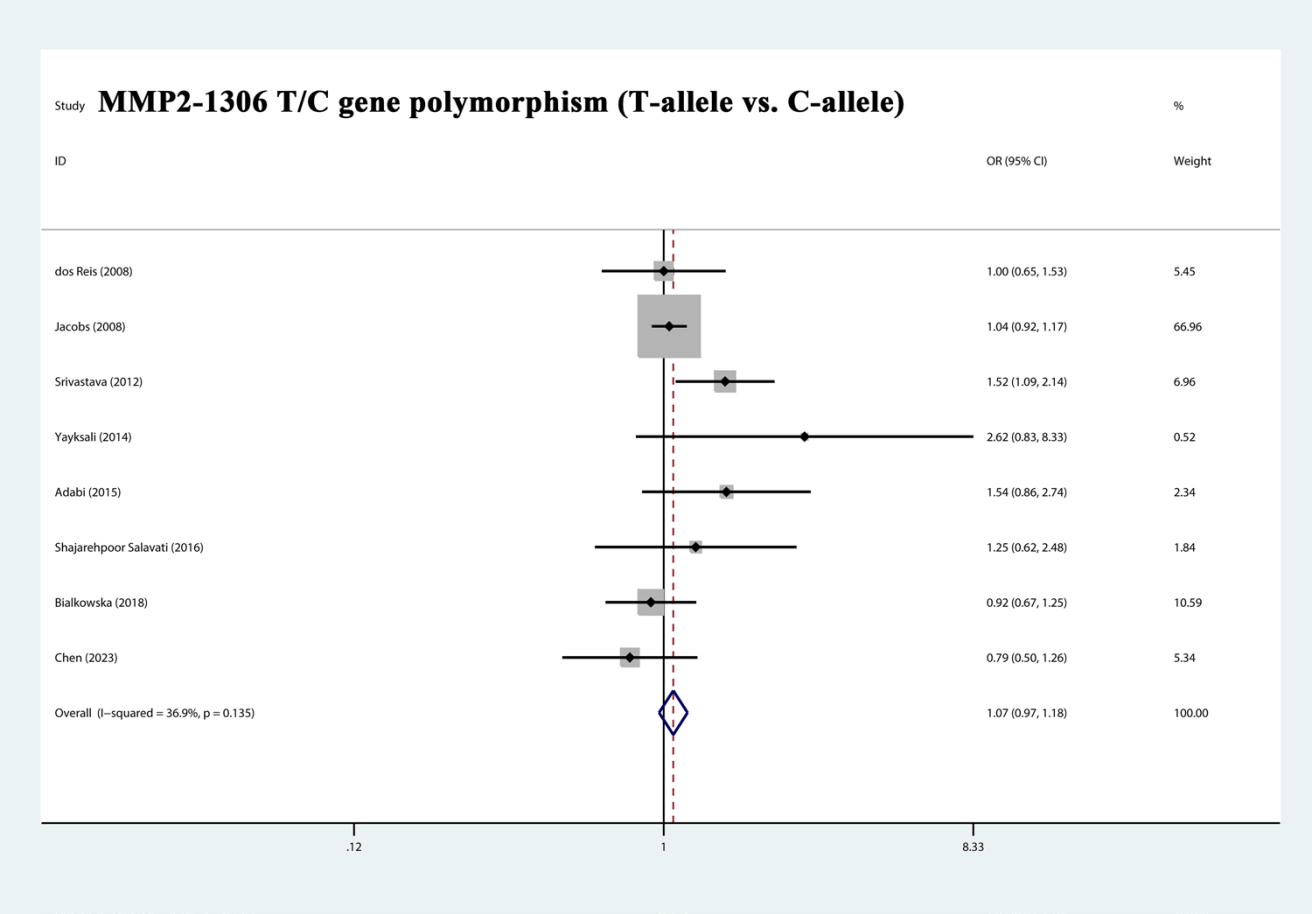
Forest plot about the relationship between the risk of PCa and the MMP2-1306 T/C gene polymorphism (T-allele vs. C-allele). There were significantly association between MMP2-1306 T/C gene polymorphism and PCa susceptibility. The squares and horizontal lines correspond to the study specific OR and 95% CI. The area of the squares reflects the weight (inverse of the variance). The diamond represents the summary OR and 95% CI.

**Figure 4:**
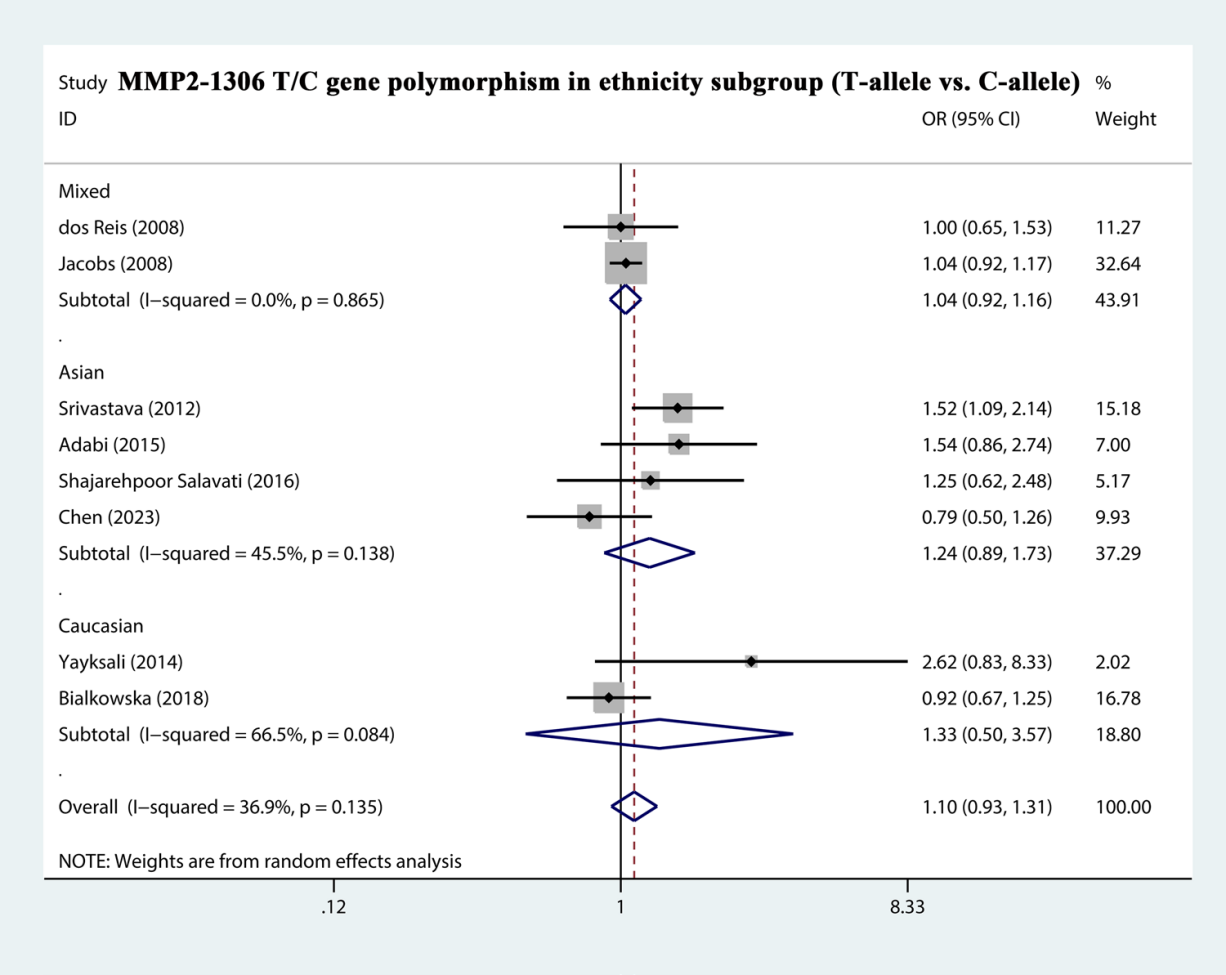
Forest plot about the relationship between the risk of PCa and the MMP2-1306 T/C gene polymorphism in ethnicity subgroup (T-allele vs. C-allele). There were significantly association between MMP2-1306 T/C gene polymorphism in ethnicity subgroup (Asian) and PCa susceptibility. The squares and horizontal lines correspond to the study specific OR and 95% CI. The area of the squares reflects the weight (inverse of the variance). The diamond represents the summary OR and 95% CI.

**Figure 5:**
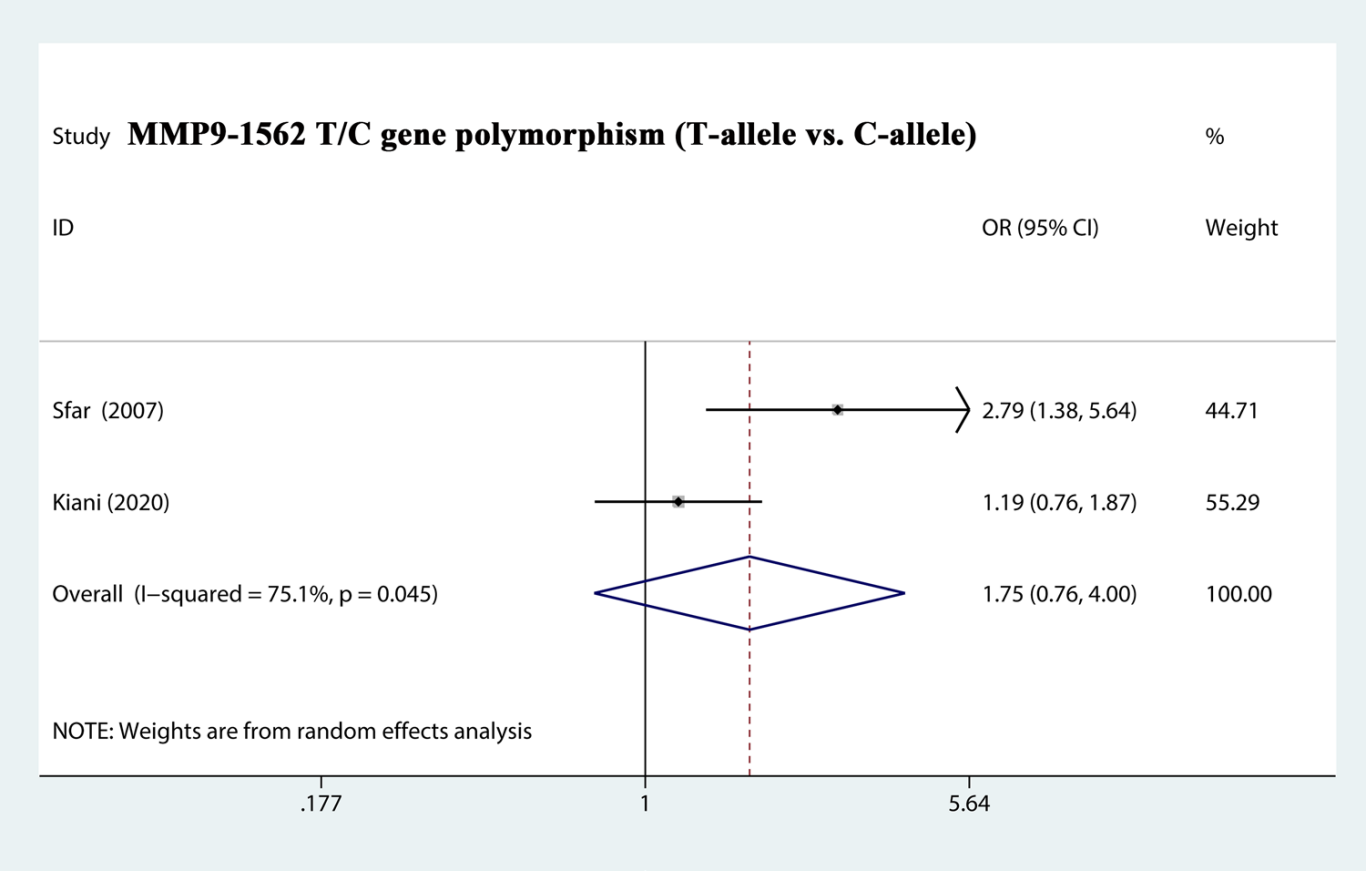
Forest plot about the relationship between the risk of PCa and the MMP9-1562 T/C gene polymorphism (T-allele vs. C-allele). There were significantly association between MMP9-1562 T/C gene polymorphism and PCa susceptibility. The squares and horizontal lines correspond to the study specific OR and 95% CI. The area of the squares reflects the weight (inverse of the variance). The diamond represents the summary OR and 95% CI.

The total risks of 3323 cases and 4043 controls of MMP1-1607 2G/1G, MMP2-1306 T/C, MMP2-735 T/C, MMP7-181 G/A, and MMP9-1562 T/C polymorphisms are summarized in Table 2. The analysis showed that MMP2-1306 T/C showed a strong association with susceptibility (T-allele vs. C-allele, OR = 1.068, 95% CI = 0.969–1.177, *P*_heterogeneity_ = 0.135, *P* = 0.186) (Fig. 3), especially in Asian Subgroup (T-allele vs. C-allele, OR = 1.257, 95% CI = 1.000–1.581, *P*_heterogeneity_ = 0.138, *P* = 0.050) (Fig. 4). Similarly, MMP9-1562 T/C polymorphisms also had the association with PCa susceptibility (T-allele vs. C-allele, OR = 1.746, 95% CI = 0.761–4.002, *P*_heterogeneity_ = 0.045, *P* = 0.188) (Fig. 5).

**Table 2:** Stratified analysis of polymorphisms of MMP genes on prostate cancer susceptibility.

Variables	No. of studies	Case/Controls	M-allele vs. W-allele	MM vs. WW	MW vs. WW	MM + MW vs. WW	MM vs. MW + WW
			OR (95%CI) *P*_h_ *P*	OR (95%CI) *P*_h_ *P*	OR (95%CI) *P*_h_ *P*	OR (95%CI) *P*_h_ *P*	OR (95%CI) *P*_h_ *P*
MMP1-1607							
Total	5	853/1027	0.801 (0.614–1.045)0.010 0.103	0.727 (0.475–1.114)0.063 0.143	1.002 (0.776–1.294)0.809 0.987	0.900 (0.713–1.137)0.521 0.376	0.691 (0.451–1.058)0.003 0.089
Ethnicity							
Asian	2	501/687	0.995 (0.839–1.181)0.726 0.958	1.010 (0.720–1.417)0.989 0.956	0.959 (0.686–1.339)0.405 0.805	0.976 (0.717–1.329)0.649 0.879	1.005 (0.790–1.279)0.390 0.965
Caucasian	2	252/240	0.814 (0.628–1.055)0.823 0.120	0.685 (0.416–1.130)0.737 0.138	1.157 (0.738–1.814)0.672 0.525	0.939 (0.625–1.410)0.887 0.762	0.622 (0.407–0.951)0.836 0.028
Mixed	1	100/100	0.439 (0.290–0.664)0.000 0.000	0.257 (0.109–0.609)0.000 0.002	0.801 (0.343–1.870)0.000 0.608	0.465 (0.211–1.024)0.000 0.057	0.303 (0.167–0.547)0.000 0.000
Source of control							
HB	3	656/830	0.706 (0.390–1.278)0.002 0.250	0.616 (0.257–1.478)0.021 0.278	0.875 (0.606–1.265)0.744 0.479	0.811 (0.585–1.125)0.306 0.210	0.622 (0.254–1.524)0.001 0.299
PB	2	197/197	0.897 (0.743–1.083)0.421 0.258	0.820 (0.556–1.210)0.280 0.318	1.136 (0.796–1.619)0.952 0.482	1.001 (0.718–1.397)0.726 0.995	0.790 (0.599–1.043)0.185 0.096
MMP2-1306							
Total	8	2336/2621	1.068 (0.969–1.177)0.135 0.186	1.041 (0.812–1.334) 0.644 0.751	1.257 (0.980–1.612)0.049 0.072	1.108 (0.984–1.248)0.119 0.091	0.981 (0.770–1.251)0.516 0.879
Ethnicity							
Asian	4	560/829	1.257 (1.000–1.581) 0.138 0.050	1.366 (0.696–2.682)0.709 0.365	1.360 (0.862–2.146)0.074 0.186	1.323 (0.867–2.018)0.083 0.194	1.205 (0.617–2.355)0.774 0.585
Caucasian	2	258/243	1.331 (0.495–3.575) 0.084 0.571	0.917 (0.455–1.848)0.191 0.809	1.020 (0.687–1.515)0.582 0.920	1.019 (0.701–1.481) 0.234 0.921	0.910 (0.460–1.802) 0.200 0.787
Mixed	2	1518/1549	1.036 (0.923–1.162) 0.865 0.548	1.012 (0.759–1.351)0.286 0.933	1.424 (0.679–2.987)0.026 0.350	1.067 (0.925–1.231)0.278 0.373	0.807 (0.405–1.609) 0.086 0.543
Source of control							
HB	5	1731/1788	1.067 (0.955–1.192)0.362 0.253	1.032 (0.781–1.362)0.624 0.826	1.489 (0.995–2.228)0.078 0.053	1.111 (0.969–1.273)0.312 0.132	0.973 (0.742–1.275) 0.342 0.840
PB	3	605/833	1.050 (0.713–1.547)0.034 0.804	1.079 (0.621–1.876)0.287 0.787	1.082 (0.699–1.676)0.057 0.724	1.078 (0.684–1.700)0.035 0.746	1.018 (0.591–1.753) 0.433 0.949
MMP7-181							
Total	3	515/733	0.980 (0.638–1.506)0.017 0.926	1.253 (0.488–3.215)0.019 0.639	0.977 (0.725 -1.314)0.177 0.875	0.968 (0.603–1.555) 0.067 0.894	1.210 (0.553–2.647) 0.035 0.633
MMP2-735							
Total	2	408/636	1.071 (0.853–1.344)0.601 0.554	1.360 (0.732–2.526)0.700 0.331	0.999 (0.755–1.323)0.366 0.997	1.039 (0.796–1.357)0.444 0.777	1.348 (0.731–2.485)0.615 0.340
MMP9-1562							
Total	2	213/256	1.746 (0.761–4.002)0.045 0.188	5.829 (0.973–34.936)0.945 0.054	1.580 (0.623–4.007)0.042 0.336	1.712 (0.680–4.309)0.041 0.253	5.457 (0.912–32.642)0.987 0.063

*P*_h_: value of *Q*-test for heterogeneity test; *P*: *Z*-test for the statistical significance of the OR; HB: hospital-based; PB: population-based; W/M: Wild/ Mutant.

The polymorphisms from MMP1-1607 2G/1G, MMP2-735 T/C and MMP7-181 G/A had no correlations with PCa risk, such as (T-allele vs. C-allele, OR = 0.801, 95% CI = 0.614–1.045, *P*_heterogeneity_ = 0.010, *P* = 0.103), (TC vs. CC, OR = 0.999, 95% CI = 0.755–1.323, *P*_heterogeneity_ = 0.366, *P* = 0.997), (GA vs. AA, OR = 0.977, 95% CI = 0.725 -1.314, *P*_heterogeneity_ = 0.177, *P* = 0.875).

## Discussion

Genetics is an important part of prostate cancer development^33,34^. But the exact genetic cause that underpins prostate cancer is not fully understood. Some studies have shown that gene polymorphisms play an important role in the risk of PCa and mutations are important drivers of altered gene expression. Furthermore, Genetic polymorphism refers to different sequence variations of the same gene in different individuals. This variation may affect the expression level of genes, resulting in differences in gene expression levels among individuals. Genetic polymorphism can affect the rate of protein synthesis, as well as gene transcription, translation, and stability. Therefore, genetic polymorphism can influence the entire process of gene expression regulation^35–38^. Therefore, studying polymorphisms in different genes remains an important direction in the field of oncology.

MMPs are a class of extracellular matrix-degrading enzymes that participate in physiological processes such as tumor metastasis and angiogenesis^7,39^. Increased expression of MMPs has been linked to tumor invasion and metastasis. The aggressiveness of tumor cells is closely related to their ability to induce MMPs production and reduce the ECM and basement membrane^40^. Furthermore, there had been several studies on MMPs genes polymorphisms and the risk of PCa in recent year^15–28^. Based on these studies and the results of our systematic analysis, MMPs are expected to become a recognized target for PCa therapy.

This meta-analysis involving 3323 cases and 4043 controls was designed to investigate the potential association among five polymorphisms of the MMPs genes and PCa risk. After meta-analysis, we found that MMP2-1306 T/C, and MMP9-1562 T/C had strong associations with PCa risk. The polymorphisms from MMP1-1607 2G/1G, MMP2-735 T/C and MMP7-181 G/A had no correlations with PCa risk. This suggested that the polymorphisms of these genes may be one of the predictors for the development of PCa. Most previous studies focused on the association of a particular polymorphism with the risk of PCa and included analyses with incomplete polymorphisms and limited data analysis^41–45^. Our study presents several advantages over previous research. Firstly, our study employed a larger sample size, thereby providing greater statistical robustness to our findings. Secondly, our study incorporated data from the most recent studies, which guaranteed that our findings provided an up-to-date perspective. Finally, the most important merit of our study was that this was the first study to examine the association of MMP1-1607 2G/1G, MMP2-1306 T/C, MMP2-735 T/C, MMP7-181 G/A and MMP9-1562 T/C polymorphisms with PCa risk using meta-analysis. This comprehensive approach provided a more nuanced understanding of genetic factors involved in MMPs and the development of PCa than previous studies.

Prostate-specific antigen (PSA) remains the cornerstone of PCa screening^46^. When PSA levels persistently increase, a transrectal ultrasound-guided prostate core needle biopsy is often performed for cancer detection. While PSA is highly specific for PCa diagnosis, some patients still have false positives. Screening for MMPs polymorphisms could be integrated into current PSA screening protocols. It is expected to increase the diagnostic positivity rate. Furthermore, this method holds promise to guide future clinical treatments and interventions aimed at preventing disease progression in susceptible populations.

In current study, we focused on the subtypes of MMP1, MMP2, MMP7, and MMP9. Other studies explored the mechanism among MMPs and the development of PCa. The Notch3-MMP3 axis in human PCa bone metastasis can block osteoclast differentiation to promote the formation of osteoblastic lesions^47^. This showed that MMP3 has a certain role in the tumor microenvironment of PCa. In the future, we can also focus on basic research between MMPs subtypes and PCa, which can deepen our understanding of MMPs polymorphisms and the specific mechanism of PCa.

The results of this meta-analysis had some limitations. After our comprehensive literature search, the number and sample size of the included studies remained relatively small, particularly MMP2-735 C/T and MMP9-1562 C/T without stratified analysis. Furthermore, due to limited information from the included studies, we were unable to detect the effects of the genetic environment. It is well known that genetic and environmental factors have a huge influence on PCa. Therefore, larger case–control studies and more nuanced studies are needed to validate the relationship between MMPs polymorphisms and prostate cancer susceptibility.

## Conclusion

The present comprehensive meta-analysis suggested that the MMP2-1306 T/C, and MMP9-1562 T/C had strong associations with PCa risk.

### Supplementary Information

Supplementary Information.

## Data Availability

The datasets presented in this study are available from online repositories. The names of the repository/repositories and accession number (s) can be found in the article/Supplementary Material.
