# Supplementary material for: Current evidence on the relationships among five polymorphisms in the matrix metalloproteinases genes and prostate cancer risk
Source: Sci Rep. 2024 May 18;14:11355. doi: 10.1038/s41598-024-62016-z (PMC11102503; doi:10.1038/s41598-024-62016-z)
Supplement: Supplementary file 1 — Supplementary Information. [file 41598_2024_62016_MOESM1_ESM.docx]

**Supplementary Table 1** Characteristics of studies included in the meta-analysis

| Author | Year | Origin | Ethnicity | Source | Case | Control | Gene | Polymorphisms | Method |
| --- | --- | --- | --- | --- | --- | --- | --- | --- | --- |
| Albayrak et al. [1] | 2007 | Turkey | Caucasian | HB | 55 | 43 | MMP-1 | -1607 1G/2G | PCR-RFLP |
| Sfar et al. [2] | 2007 | Tunisia | African | PB | 101 | 106 | MMP-9 | -1562 C/T | PCR-RFLP |
| Dos Reis et al. [3] | 2008 | Brazil | Mixed | HB | 100 | 100 | MMP-1  MMP-2  MMP-7 | -1607 1G/2G  -1306 C/T  -181 A/G | Taqman |
| Jacobs et al. [4] | 2008 | USA | Mixed | HB | 1418 | 1449 | MMP-2 | -1306 C/T | MassARRAY |
| Tsuchiya et al. [5] | 2009 | Japan | Asian | PB | 283 | 251 | MMP-1 | -1607 1G/2G | PCR |
| Srivastava et al. [6] | 2012 | India | Asian | PB | 190 | 200 | MMP-2 | -735 C/T  -1306 C/T | PCR-RFLP |
| Yaykaşli et al. [7] | 2014 | Turkey | Caucasian | HB | 61 | 46 | MMP-2 | -1306 C/T | PCR-RFLP |
| Adabi et al. [8] | 2015 | Iran | Asian | HB | 102 | 139 | MMP-2 | -1306 C/T | PCR-RFLP |
| Shajarehpoor Salavati  et al. [9] | 2016 | Iran | Asian | HB | 50 | 54 | MMP-2 | -1306 C/T | HRM |
| Białkowska et al. [10] | 2018 | Poland | Caucasian | PB | 197 | 197 | MMP-1 MMP-2  MMP-7 | -1607 1G/2G  -1306 C/T  -181 A/G | Taqman |
| Liao et al. [11] | 2018 | China | Asian | PB | 218 | 436 | MMP-1 | -1607 1G/2G | PCR-RFLP |
| Kiani et al. [12] | 2020 | Iran | Asian | HB | 112 | 150 | MMP-9 | -1562 C/T | PCR-RFLP |
| Chen et al. [13] | 2020 | China | Asian | PB | 218 | 436 | MMP-2 | -735 C/T  -1306 C/T | PCR-RFLP |
| Liao et al. [14] | 2023 | China | Asian | PB | 218 | 436 | MMP-7 | -181 A/G | PCR-RFLP |

HB: hospital-based; PB: population-based; SOC; source of control; PCR-RFLP: polymerase chain reaction followed by restriction fragment length polymorphism; PCR: polymerase chain reaction; MassARRAY: matrix assisted laser desorption/ionization time of flight mass spectrometry

Reference:

1. Albayrak S, Cangüven Ö, Göktaş C, Aydemir H, Köksal V. Role of MMP-1 1G/2G Promoter Gene Polymorphism on the Development of Prostate Cancer in the Turkish Population. Urol Int. 2007;79:312–5.

2. Sfar S, Saad H, Mosbah F, Gabbouj S, Chouchane L. TSP1 and MMP9 genetic variants in sporadic prostate cancer. Cancer Genet Cytogenet. 2007;172:38–44.

3. Dos Reis ST, Villanova FE, De Andrade PM, Pontes J, Silva IA, Canavez FC, et al. Polymorphisms of the matrix metalloproteinases associated with prostate cancer. Mol Med Rep. 2008;1:517–20.

4. Jacobs EJ, Hsing AW, Bain EB, Stevens VL, Wang Y, Chen J, et al. Polymorphisms in angiogenesis-related genes and prostate cancer. Cancer Epidemiol Biomarkers Prev. 2008;17:972–7.

5. Tsuchiya N, Narita S, Kumazawa T, Inoue T, Ma Z, Tsuruta H, et al. Clinical significance of a single nucleotide polymorphism and allelic imbalance of matrix metalloproteinase-1 promoter region in prostate cancer. Oncol Rep. 2009;22:493–9.

6. Srivastava P, Lone TA, Kapoor R, Mittal RD. Association of Promoter Polymorphisms in MMP2 and TIMP2 with Prostate Cancer Susceptibility in North India. Archives of Medical Research. 2012;43:117–24.

7. Yaykaşli KO, Kayikçi MA, Yamak N, Soğuktaş H, Düzenli̇ S, Arslan AO, et al. Polymorphisms in MMP-2 and TIMP-2 in Turkish patients with prostate cancer. Turk J Med Sci. 2014;44:839–43.

8. Adabi Z, Mohsen Ziaei SA, Imani M, Samzadeh M, Narouie B, Jamaldini SH, et al. Genetic Polymorphism of MMP2 Gene and Susceptibility to Prostate Cancer. Archives of Medical Research. 2015;46:546–50.

9. Shajarehpoor Salavati L, Tafvizi F, Manjili HK. The association between MMP2 -1306 C > T (rs243865) polymorphism and risk of prostate cancer. Ir J Med Sci. 2017;186:103–11.

10. Białkowska K, Marciniak W, Muszyńska M, Baszuk P, Gupta S, Jaworska-Bieniek K, et al. Association of zinc level and polymorphism in MMP-7 gene with prostate cancer in Polish population. PLoS One. 2018;13:e0201065.

11. Liao C-H, Wu H-C, Hu P-S, Hsu S-W, Shen T-C, Hsia T-C, et al. The Association of Matrix Metalloproteinase-1 Promoter Polymorphisms with Prostate Cancer in Taiwanese Patients. Anticancer Res. 2018;38:3907–11.

12. Kiani A, Kamankesh M, Vaisi-Raygani A, Moradi M-R, Tanhapour M, Rahimi Z, et al. Activities and polymorphisms of MMP-2 and MMP-9, smoking, diabetes and risk of prostate cancer. Mol Biol Rep. 2020;47:9373–83.

13. Chen L-H, Chiu K-L, Hsia T-C, Lee Y-H, Shen T-C, Li C-H, et al. Significant Association of MMP2 Promoter Genotypes to Asthma Susceptibility in Taiwan. In Vivo. 2020;34:3181–6.

14. Liao C-H, Chang W-S, Hsu W-L, Hu P-S, Wu H-C, Hsu S-W, et al. Association of Matrix Metalloproteinase-7 Genotypes With Prostate Cancer Risk. Anticancer Research. 2023;43:381–7.
